# Supplementary material for: Complexome profiling of the Chlamydomonas psb28 mutant reveals TEF5 as an early PSII assembly factor
Source: Plant Cell. 2025 Mar 17;37(6):koaf055. doi: 10.1093/plcell/koaf055 (PMC12164590; doi:10.1093/plcell/koaf055)
Supplement: koaf055_Supplementary_Data [file koaf055_supplementary_data.zip › psb28_interactive.zip!/plots/intensity/qualityPlots/peptides/_Cre05.g242350.t1.2.html]

### Cre05.g242350.t1.2

Trivial name:   
SeqCoverage [median]: 13.40  
Mass: 113.98 kDa  
  
 

| Sequence | MissedCleavage | Mass [Da] | wtIntensity | muIntensity | Score |
| --- | --- | --- | --- | --- | --- |
| ELQTETAHVTR | 0 | 1283.65 | 477.49 | 1047.75 | 51.25 |
| IQEQISAAAKEDDDTPLKK | 2 | 2099.07 | 0.00 | 1724.47 | 78.02 |
| LADIGVAMGIAGTEVAK | 0 | 1614.87 | 0.00 | 587.24 | 53.25 |
| LGVADPAETAAAR | 0 | 1240.64 | 729.13 | 662.74 | 67.11 |
| LLHANTGSDASR | 0 | 1240.62 | 0.00 | 1250.20 | 100.04 |
| LLKEQGEVTAMTGDGVNDAPALK | 1 | 2356.19 | 0.00 | 861.41 | 46.58 |
| MVSDLPSR | 0 | 903.45 | 0.00 | 599.05 | 93.37 |
| SMSVLVRPAGAAR | 1 | 1313.72 | 0.00 | 483.45 | 44.54 |
| VEQASLTGESVAVNK | 0 | 1530.79 | 0.00 | 80584.77 | 58.37 |
|  |  |  | 1206.6 | 87801.1 |  |
